# Differentiated thyroid carcinoma: a 5-years survival study at a referral hospital in Brazil

**DOI:** 10.11606/S1518-8787.2019053001496

**Published:** 2019-11-21

**Authors:** Anne Karin da Mota Borges, Jeniffer Dantas Ferreira, Sergio Koifman, Rosalina Jorge Koifman

**Affiliations:** I Instituto Nacional de Câncer José Alencar Gomes da Silva. Coordenação de Prevenção e Vigilância. Divisão de Vigilância e Análise de Situação; II Instituto Nacional de Câncer José Alencar Gomes da Silva. Coordenação de Prevenção e Vigilância. Divisão de Vigilância e Análise de Situação

**Keywords:** Thyroid Neoplasms, epidemiology, Carcinoma, Survival Rate, Prognosis, Cohort Studies

## Abstract

**BACKGROUND:**

Although the prognosis of differentiated thyroid carcinoma (DTC) therapy is considered excellent over time, some cases have a poorer prognosis and evolve into death.

**OBJECTIVE:**

This study aimed to estimate the 5-year specific survival and to identify prognosis factors in a cohort of DTC adult subjects.

**METHODS:**

Survival probability was estimated by Kaplan-Meier’s method in a retrospective hospital-based cohort study. Comparisons were made by log-rank test. Prognosis factors were identified using Cox risk modeling and crude and adjusted Hazard Ratio measures were obtained. Two models were estimated, considering age grouping of the 7^th^ and 8^th^ editions of TNM.

**RESULTS:**

Specific 5-year survival in the cohort was 98.5% (95%CI: 94.2 – 97.5). Considering TNM 7^th^ edition, the risk estimates were 9.88 (95%CI: 1.67 – 58.33) for age group ≥ 55 years, 18.87 (95%CI: 7.38 – 48.29) for individuals with distant metastasis, 6.36 (95%CI: 2.26 – 17.91) for patients who underwent lymphadenectomy and 0.16 (95%CI: 0.06 – 0.43) for those who received radioiodine therapy. For TNM 8^th^ edition, the risk estimates were 10.12 (95%CI: 2.05 – 50.09) for age group ≥ 55 years, 12.43 (95%CI: 4.58 – 33.77) for individuals with distant metastasis, 5.06 (95%CI: 1.82 – 14.05) for patients who underwent lymphadenectomy and 0.19 (95%CI: 0.07 – 0.51) for those who received radioiodine therapy.

**CONCLUSIONS:**

This cohort had a very high survival over a 5-year period. The prognosis was negatively influenced by age, distant metastasis and lymphadenectomy, whereas radioiodine therapy was found to be protective.

## INTRODUCTION

Thyroid cancer (TC) is considered the most common malignant neoplasm of the endocrine system, accounting for 3.1% of all cancers^1^^–^^3^. Its incidence has soared out considerably in the last three decades^4^^–^^8^. Mortality on the other hand, besides being low^1^, has shown a declining trend^9^^,^^10^. The difference observed between incidence and mortality magnitudes is possibly due to the higher frequency of the disease occurring in histological types with a good prognosis, specifically differentiated thyroid carcinomas (DTC) – which include papillary thyroid carcinoma (PTC) and follicular thyroid carcinoma (FTC)^3^^,^^9^^,^^11^. Although the outcome of effective treatment is excellent in the long-term, a small portion of the patients present recurrence and some eventually do not respond to conventional therapy, evolving to death^3^^,^^11^^,^^12^. Identifying potentially aggressive disease cases in advance and evaluating the most appropriate treatment strategy for each case has been a challenge.

Prospective randomized controlled trials would be suitable for identifying factors that predict prognosis for DTC in the long-term, especially those related to treatment. However, the low mortality rate of the disease combined with its prolonged nature and follow-up costs make it difficult to conduct these trials. Therefore, there are few studies in the scientific literature that explore empirical data on this theme, mainly in Brazilian cohorts, justifying observational studies. Thus, the objectives of this study were to estimate the specific 5-year survival and to identify the prognostic factors of a hospital cohort of adult patients with DTC.

## MATERIAL AND METHODS

### Study Design and Population

This retrospective hospital-based cohort study was conducted at the Brazilian National Cancer Institute José Alencar Gomes da Silva (INCA), and the outcome variable was the time between the diagnosis of primary DTC and the death due to the disease. The study population consisted of patients who were attended between January 1^st^, 2000 and December 31, 2010 and registered in the database of the Hospital-based Cancer Registry (HBCR). Eligibility was considered as histologically confirmed cases of thyroid carcinoma according to the International Classification of Disease for Oncology – Third Edition, site codes (C73.0 – 73.9), and who underwent a therapeutic plan and follow-up at INCA (N = 739); who had a diagnosis at or above 20 years of age (N = 672), and presented the following histological codes (N = 620): 8050, 8260, 8340-8344, and 8350 (PTC), and 8290, 8330-8332, and 8335 (FTC).

Cases (N = 51) with a history of other primary tumor or previous TC diagnosis were excluded from the study. After reviewing all the histopathological reports, the following cases were excluded: PTC recurrence (N = 2); adenocarcinoma no further specification (N = 2); unclear diagnostic about primary or metastatic tumor (N = 1); primary pulmonary tumor (N = 1); uncertainty about tumor malignancy, adenoma or carcinoma (N = 1). The sample thus consisted of 562 cases of primary DTC.

### Data Collection

Selected sociodemographic variables, and variables related to the tumor, treatment and follow-up were collected from HBCR database. To complete missing data and to update follow-up information, the medical records of the patients were evaluated.

For the cases lost during follow-up period, the surveillance of deaths with their respective dates and basic causes was conducted by consulting the database of the Mortality Information System (SIM) of the state of Rio de Janeiro. Other public databases were consulted (Individual Taxpayer Registration − CPF and Superior Electoral Court − TSE) for cases with information unavailable on SIM.

### Covariates of Interest

The variables under study were: sociodemographic (gender and age at diagnosis); tumor characteristics (tumor size, stage, histological type, regional lymph node metastasis, and distant metastasis); and treatment (type of surgery, unilateral or bilateral, cervical and/or mediastinal lymphadenectomy, radioiodine, and radiotherapy). Due to the high percentage of lack of information for the clinical staging of the tumor (66.3%), pathological staging (pTNM) was used for surgical cases and clinical staging for non-surgical patients (0.5%), based on the 7^th^ and 8^th^ editions of the TNM staging system^13^^,^^14^. One of the differences between TNM 7^th^ and 8^th^ editions is the change in the age of poor prognosis from 45 to 55 years. Moreover, the T3 definition has been revised. Finally, there were changes in groupings by stages for the age group ≥ 55 years.

### Statistical Analysis

Baseline characteristics were reported using measures of central tendency and dispersion for continuous variables and proportions for categorical variables. Descriptive data were compared across groups using Student’s t-test for continuous variables and Chi-square or Fisher’s exact tests for categorical variables. In both tests, the significance level was less than 0.05.

Specific survival was defined as the duration from the date of diagnosis to death due to DTC. Survival time was censored for patients alive at the end of the study period, lost during follow-up or deceased due to other causes. The Kaplan-Meier method was used to compute 5-year survival probability and its respective 95% confidence interval (CI). The survival curves were plotted to present the differences in the survival of the patients according to the effective categorical variables, and survival distributions were compared across groups using the log-rank test. Statistical significance was accepted as p < 0.05.

Multivariate survival analysis and hazard ratio (HR) were calculated using Cox’s model. Two models were estimated, considering age grouping of TNM 7^th^ and 8^th^ editions. A multivariate model was constructed for each one by adding each of the variables that, in a univariate analysis, presented p ≤ 0.20 in Wald’s test, starting from the lowest to the highest p-value. At each step, the variables that presented p ≥ 0.10 were excluded. In the final model, significant variables were maintained at 5% level – except for gender and age at diagnosis, which were kept independent of their statistical significance due to their potential for confounding.

For DTC, staging was composed by age at diagnosis and the characteristics related to the size of the primary tumor, the regional lymph nodes, and the distant metastasis. Thus, instead of the staging itself, it was decided to use its components in a disaggregated way to evaluate the effect of each covariate on DTC survival. To compare these covariates, cases with missing values were excluded from Cox’s model. The final model was chosen based on the likelihood ratio test (ANOVA) at a significance level of 0.05. We looked for interactions.

The overall fit of the model was evaluated by the explanatory power (R^2^ of the selected model/R^2^ of the saturated model) and concordance probability. The assumption of Cox’s proportional hazards model was assessed by the analysis of Schoenfeld residues and the impact of outliers was analyzed using martingale, deviance and score residuals. The data was analyzed by R software, version 3.4.0, using the Survival package.

### Ethical Aspects

In compliance with the requirements of Resolution 466/2012 and complements of the National Board of Health, this study was approved by the Research Ethics Committee of the National School of Public Health Sergio Arouca, under protocol CAAE no. 62062116.8.0000.5240 and, by INCA Committee, protocol CAAE no. 57282216.8.0000.5274.

## RESULTS

### Cohort characteristics

Of the 562 cases of DTC, 79.0% were women, for 3.8 female to male ratio (F/M). The mean age at diagnosis was 46.5 years (median age 45 years, ranging from 20 to 91 years). The mean primary tumor size, known for 85.9% of cases, was 2.3 cm (median of 1.8 cm, ranging from 0.1 to 14.0 cm). Lymph node metastasis, with information for 98.2% of the cohort, and distant metastasis were present in 38.8% and 5.3% of the patients, respectively. Most of the cases were of staging I (61.2% and 74.8% considering TNM 7^th^ and 8^th^ editions, respectively).

Compared with females, males exhibited a larger mean tumor size (3.2 cm versus 2.1 cm, p < 0.001), a lower proportion of microcarcinomas, i.e., tumors ≤ 1.0 cm (21.0% versus 33.1%, p = 0.023) and a higher proportion of regional lymph node metastasis (50.9% versus 35.6%, p = 0.004). No statistically significant differences were found for age at diagnosis, distant metastasis and staging between women and men.

The cohort was composed of 91.3% of cases of PTC. Compared with PTC, FTC showed a higher mean age at diagnosis (54.4 years versus 46.0 years, p = 0.015) and higher mean tumor size (3.0 cm versus 2.3 cm, p = 0.021). The F/M ratio was 4.1 for PTC and 1.7 for FTC.

Table 1 shows the characteristics of the study population by histological types. FTC presented a higher proportion of men and a higher frequency of patients who were positive for distant metastasis. In contrast, PTC showed a higher proportion of cases with regional lymph node metastasis and a higher frequency of microcarcinomas. The distribution of staging differed between histological types, while PTC presented a higher proportion of staging I, FTC exhibited higher frequencies of stages II, III and IVC (TNM 7^th^ edition) or II, III and IVB (TNM 8^th^ edition).

**Table 1:** Sociodemographics and clinical characteristics of patients diagnosed with thyroid cancer by histological type, Brazil, 2000-2015.

Variables^a^		Histological types				p value^b^
		Papillary		Follicular		
		N	%	N	%	
**Gender**						
	Male	100	19.5	18	36.7	**0.008**
	Female	413	80.5	31	63.3	
	Total	513	100	49	100	
**Age at diagnosis** − **TNM 7**^**th**^ **edition**						
	20-44	251	48.9	14	28.6	**0.010**
	≥ 45	262	51.1	35	71.4	
	Total	513	100	49	100	
**Age at diagnosis** − **TNM 8**^**th**^ **edition**						
	20-54	385	75.0	29	59.2	**0.025**
	≥ 55	128	25.0	20	40.8	
	Total	513	100	49	100	
**Staging** − **TNM 7**^**th**^ **edition**						
	I	320	63.7	17	34.7	**< 0.00**1
	II	43	8.6	6	12.2	
	III	59	11.8	13	26.5	
	IVA	61	12.2	6	12.2	
	IVB	3	0.6	−	−	
	IVC	16	3.2	7	14.3	
	Total	502	100	49	100	
**Staging** − **TNM 8**^**th**^ **edition**						
	I	387	77.1	25	51.0	**< 0.001**
	II	66	13.1	13	26.5	
	III	30	6.0	4	8.2	
	IVA	3	0.6	−	−	
	IVB	16	3.2	7	14.3	
	Total	502	100	49	100	
**Tumor size**						
	< 4.0 cm	367	82.3	27	73.0	0.237
	≥ 4.0 cm	79	17.7	10	27.0	
	Total	446	100	37	100	
**Microcarcinoma**						
	No	303	67.9	33	89.2	**0.005**
	Yes	143	32.1	4	10.8	
	Total	446	100	37	100	
**Regional lymph nodes metastasis**						
	No	294	58.4	44	89.8	**< 0.001**
	Yes	209	41.6	5	10.2	
	Total	503	100	49	100	
**Distant metastasis**						
	No	491	95.7	41	83.7	**0.003**
	Yes	22	4.3	8	16.3	
	Total	513	100	49	100	

a Missings: staging (N = 11; 1.8%), tumor size (N = 79; 14.1%), regional lymph nodes metastasis (N = 10, 1.8%) e microcarcinoma (N = 79; 14.1%).

b p value of Chi-square or Fisher’s exact tests, being in bold when p < 0.05.

Multifocal lesions were only observed for PTC (10.7%). The most commonly observed PTC variants were classical (25.3%) and follicular (17.7%) variants, but no histological subtype specification was found for 53.8% of the cases. For FTC, 36.7% of the patients who had the variant information belonged to the Hürthle cell subtype.

### Follow-up

During the 5-year follow-up, 539 censures and 23 deaths related to TC were observed. Censored group consisted of 522 living individuals at the end of 60 months, 14 deaths from other causes and 3 follow-up losses (0.5%), which were female, PTC, stages I, III and IVA (TNM 7^th^ edition) or I, II, III (TNM 8^th^ edition) and follow-up times of 34.5, 8.2 and 6.7 months, respectively. Moreover, they were older (mean age 66.3 years versus 46.4 years, p = 0.018) when compared to complete follow-up cases. The mean follow-up time of the cohort was 57.5 months, being 58.9 months for the censored cases and 23.4 months for those who suffered the outcome.

### Deaths related to Differentiated Thyroid Cancer

Of the 23 deaths observed, 91.3% were aged ≥ 45 years (TNM 7^th^ edition) or 87.0% were aged ≥ 55 years (TNM 8^th^ edition) and 56.5% had distant metastasis (Table 2). All patients with FTC who died had distant metastasis. Among PTC deaths, the proportion of patients with distant metastasis was 47.4%. Compared with PTC, FTC deaths had smaller tumors (mean size 2.3 cm versus 4.2 cm, p = 0.014) and higher mean age at diagnosis (72.5 years versus 65.4 years), with no significant difference. In both histological types, 50% of the deaths had regional lymph node metastasis.

**Table 2:** Conditional 5-year survival probability estimated by Kaplan-Meier, mean survival time according to sociodemographic, tumor characteristics and treatment for patients with differentiated thyroid cancer.

Variables^a^		Cases		Deaths		Conditional 5-year survival probabilities		Log-rank^d^	Mean survival time^e^
		N	%	N	%	S(t)^b^	95% CI^c^		
**Gender**									
	Male	118	21.0	10	43.5	91.5	86.6 – 96.7	**0.009**	57.5
	Female	444	79.0	13	56.5	97.0	95.5 – 98.6		57.5
	Total	562	100	23	100				
**Age at diagnosis** − **TNM 7**^**th**^ **edition**									
	20-44	265	47.2	2	8.7	99.2	98.2 − 100	**< 0.001**	59.1
	≥ 45	297	52.8	21	91.3	92.8	89.8 – 95.8		56.0
	Total	562	100	23	100				
**Age at diagnosis** − **TNM 8**^**th**^ **edition**									
	20-54	414	73.7	3	13.0	99.3	98.5 − 100	**< 0.001**	59.1
	≥ 55	148	26.3	20	87.0	85.9	80.4 − 91.9		52.8
	Total	562	100	23	100				
**Staging** − **TNM 7**^**th**^ **edition**									
	I/II	386	70.1	2	8.7	99.5	98.8 − 100	**< 0.001**	59.1
	III	72	13.1	3	13.0	95.8	91.2 − 100		57.4
	IVA/IVB	70	12.7	6	26.1	90.8	84.0 – 98.1		53.7
	IVC	23	4.2	12	52.2	47.1	30.4 – 73.0		39.8
	Total	551	100	23	100				
**Staging** − **TNM 8**^**th**^ **edition**									
	I/II	491	89.1	7	30.4	98.6	97.5 – 99.6	**< 0.001**	58.8
	III/ IVA	37	6.7	4	17.4	87.3	76.4 – 99.7		50.0
	IVB	23	4.2	12	52.2	47.1	30.4 – 73.0		39.8
	Total	551	100	23	100				
**Tumor size**									
	< 4.0 cm	394	81.6	10	47.6	97.4	95.9 – 99.0	**< 0.001**	58.1
	≥ 4.0 cm	89	18.4	11	52.4	87.1	80.3 – 94.5		54.1
	Total	483	100	21	100				
**Distant metastasis**									
	No	532	94.7	10	43.5	98.1	96.9 – 99.3	**< 0.001**	58.4
	Yes	30	5.3	13	56.5	54.8	39.2 – 76.4		40.7
	Total	562	100	23	100				
**Lymphadenectomy**									
	No	381	67.8	11	47.8	97.1	95.4 – 98.8	**0.031**	58.3
	Yes	181	32.2	12	52.2	93.2	89.5 – 97.0		55.8
	Total	562	100	23	100				
**Radioiodine therapy**									
	No	122	21.7	13	56.5	88.8	83.2 – 94.7	**< 0.001**	53.1
	Yes	440	78.3	10	43.5	97.7	96.3 – 99.1		58.7
	Total	562	100	23	100				
**Radiotherapy**									
	No	536	95.4	16	69.6	97.0	95.5 – 98.4	**< 0.001**	58.0
	Yes	26	4.6	7	30.4	70.5	54.3 – 91.6		46.0
	Total	562	100	23	100				

a Missings: staging (N = 11; 2.0%) and tumor size (N = 79; 14.1%).

b Conditional 5-year survival probabilities.

c 95% confidence interval.

d p value of log-rank test, being in bold when p < 0.05.

e Mean survival time in months.

There were no statistically significant differences between the survival curves of histological type, microcarcinoma, multifocal lesions and regional lymph node metastasis.

### Survival

**Figure 1:**
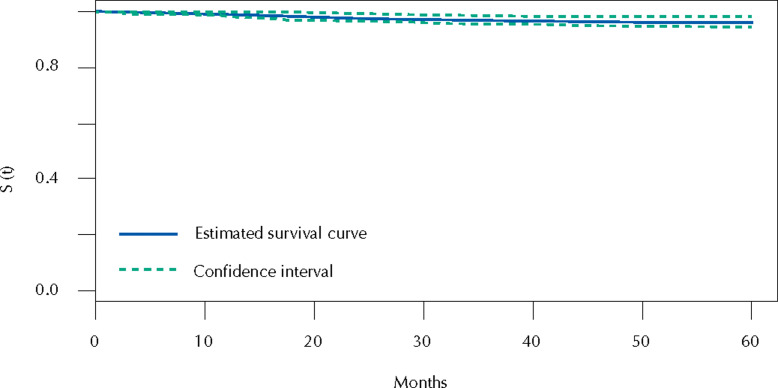
Kaplan-Meier plot of 5-year specific survival in differentiated thyroid cancer patients.

Figure 1 presents the 5-year specific survival probability of DTC, which was 95.8% (95%CI: 94.2–97.5).

**Figure 2:**
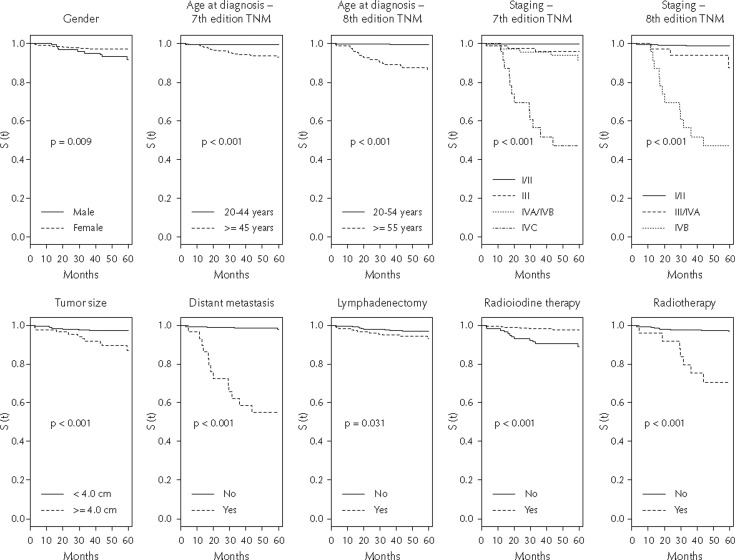
Kaplan-Meier plot of 5-year specific survival in differentiated thyroid cancer patients by sociodemographic, treatment and tumorrelated covariates.

Table 2 and Figure 2 present, respectively, the 5-year conditional survival probability and the Kaplan-Meier curves according to sociodemographic and clinical variables. Women showed greater survival (97.0%; 95%CI: 95.5 – 98.6) than men (91.5%; 95%CI: 86.6 – 96.7). A similar scenario occurred with individuals diagnosed in the age group of 20–44 years (99.2%; 95%CI: 98.2 – 100) compared to those aged ≥ 45 years (92.8%, 95%CI: 89.9 – 95.8), and with age group of 20-54 years (99.3%; 95%CI: 98.5 – 100) compared to those aged ≥ 55 years (85.9%; 95%CI: 80.4 – 91.9). There was a greater probability of survival for tumors smaller than 4.0 cm (97.4%; 95%CI: 95.9 – 99.0) than for those ≥ 4.0 cm (87.1%, 95%CI: 80.3 – 94.5). The positive individuals for distant metastasis had worse survival (54.8%; 95%CI: 39.2 – 76.4) than those negative (98.1%; 95%CI: 96.9 – 99.3). Considering TNM 7^th^ edition, stages I/II, III and IVA/IVB presented survival probabilities higher than 90.0%. On the other hand, the survival of the IVC stage was 47.1% (95%CI: 30.4 – 73.0). By the TNM 8^th^ edition, for stages I/II, III/IVA and IVB the survival probabilities were 98.6% (95%CI: 97.5 – 99.6), 87.3% (95%CI: 76.4 – 99.7) and 47.1% (95%CI: 30.4 – 73.0), respectively.

Although FTC showed lower survival (91.8%; 95%CI: 84.5 – 99.8) than PTC (96.2%; 95%CI: 94.6 – 97.9), no statistically significant difference between the estimated curves was observed (p = 0.139). Moreover, no significant differences were observed in the survival curves estimated according to the variables microcarcinoma, multifocal lesions and regional lymph nodes metastasis.

In total, 99.5% of the patients underwent surgery, 96.2% underwent total thyroidectomy and 3.8% subtotal thyroidectomy. Only three cases (0.5%) did not undergo any surgical intervention because the tumors were considered non-resectable. Thus, excluding these three cases from the analysis, no statistically significant difference was observed between total thyroidectomy and subtotal thyroidectomy survival curves.

Cases that underwent lymphadenectomy had lower survival (93.2%, 95%CI: 89.5 – 97.0) than those who did not undergo this type of intervention (97.1%, 95%CI: 95.4 – 98.8). Patients who received adjuvant radioiodine therapy had better survival (97.7%; 95%CI: 96.3 – 99.1) when compared to those who did not receive this treatment (88.8%; 95%CI: 83.2 – 94.7). Compared with cases treated with radiotherapy (70.5%, 95%CI: 54.3 – 91.6), other cases had better survival (97.0%, 95%CI: 95.5 – 98.4) (Table 2 and Figure 2).

### Prognostic factors

For both analyses – considering the age grouping of TNM 7^th^ and 8^th^ editions – the most appropriate multivariate model was one in which the effects of covariables age, distant metastasis, lymphadenectomy, and radioiodine therapy were controlled by baseline risk variation attributable to covariant sex strata (Cox’s model stratified by sex) present in Table 3. Considering the age grouping of TNM 7^th^ edition, cases diagnosed in the age group ≥ 45 years had 9.88 times greater risk of death from TC than those aged 20-44 years (95%CI: 1.67 – 58.33); the positive patients for distant metastasis showed 18.87 (95%CI: 7.38 – 48.29) higher risk of dying than the negative cases; the individuals submitted to lymphadenectomy presented 6.36 (95%CI: 2.26 – 17.91) higher risk of death than those who had no indication for this surgical intervention; and the cases of TC that received radioiodine therapy had a 84.0% lower risk of death than those who did not have a recommendation for this therapeutic modality (adjusted HR: 0.16; 95%CI: 0.06 – 0.43). Considering the age grouping of TNM 8^th^ edition, the risk estimates were 10.12 (95%CI: 2.05 – 50.09) for age group ≥ 55 years, 12.43 (95%CI: 4.58 – 33.77) for positive individuals with distant metastasis, 5.06 (95%CI: 1.82 – 14.05) for patients underwent to lymphadenectomy and 0.19 (95%CI: 0.07 – 0.51) for those who received radioiodine therapy.

**Table 3:** Crude and adjusted Hazard Ratios for differentiated thyroid cancer patients, using the Cox model.

Variables		Crude HR^a^	95%CI^b^	p value^c^	Multivariate model using age grouping of the TNM 7^th^ edition			Multivariate model using age grouping of the TNM 8^th^ edition		
					adjusted HR^a^	95%CI^b^	p value^c^	adjusted HR^a^	95%CI^b^	p value^c^
**Gender**										
	Female	1.00			Multivariate Cox model stratified by gender					
	Male	2.75	1.16 – 6.52	**0.022**						
**Age at diagnosis** – **TNM 7**^**th**^ **edition**										
	20-44	1.00			1.00			-	-	-
	≥ 45	8.68	2.02 – 37.27	**0.004**	9.88	1.67 – 58.33	**0.011**	-	-	-
**Age at diagnosis** – **TNM 8**^**th**^ **edition**										
	20-54	1.00			-	-	**-**	1.00		
	≥ 55	17.60	5.18 – 59.76	**< 0.001**	-	-	**-**	10.12	2.05 – 50.09	**0.005**
**Distant metastasis**										
	No	1.00			1.00			1.00		
	Yes	28.61	12.08 – 67.77	**< 0.001**	18.87	7.38 – 48.29	**< 0.001**	12.43	4.58 – 33.77	**< 0.001**
**Radioiodine therapy**										
	No	1.00			1.00			1.00		
	Yes	0.22	0.09 – 0.52	**0.001**	0.16	0.06 – 0.43	**< 0.001**	0.19	0.07 – 0.51	**0.001**
**Lymphadenectomy**										
	No	1.00			1.00			1.00		
	Yes	2.57	1.10 – 6.02	**0.035**	6.36	2.26 – 17.91	**0.001**	5.06	1.82 – 14.05	**0.002**
**Tumor size**										
	< 4.0 cm	1.00			-	-	-	-	-	-
	≥ 4.0 cm	5.18	2.20 – 12.2	**< 0.001**	-	-	-	-	-	-
**Microcarcinoma**										
	Yes	1.00			-	-	-	-	-	-
	No	2.71	0.80 – 9.20	0.110	-	-	-	-	-	-
**Radiotherapy**										
	No	1.00								
	Yes	8.96	3.47 – 23.13	**< 0.001**						

a Hazard Ratio;

b CI= confidence interval;

c Wald’s test p value, being in bold when p < 0.05.

Histological type and regional lymph nodes metastasis variables presented p > 0.20 in univariate Cox model.

Schoenfeld’s residues showed that the premise of proportional hazards was not violated (p = 0.132 and 0.329 for models considering age grouping of TNM 7^th^ and 8^th^ editions, respectively). Considering the age grouping of TNM 7^th^ edition, the final model explained 39.2% of the data variability and the probability of agreement was 95.9%. For the 8th edition, they were 39.7% and 95.2%, respectively. No interaction was observed between the variables. Using the martingale and deviance residues, for multivariate model considering the age grouping of TNM 7^th^ edition, three outliers were identified: a woman who died in a very short time (3.0 months), even though she was 38 years old, PTC and stage I; a 72-year-old woman with PTC and IVA staging (tumor size = 7.0 cm) who died at 23.3 months; and a 75-year-old woman with PTC and IVA staging (tumor size = 2.0 cm) who had the outcome at 11.8 months. For final model considering age grouping TNM 8^th^ edition, three outliers also were identified: the first two described previously and a 51-year-old woman with PTC and IVB staging with outcome at 36.1 months. No influential points were observed by scoring residue analysis.

## DISCUSSION

The results of this study pointed out a higher 5-year survival rate for patients with DTC. For either both sexes, diagnostic age, distant metastasis, lymphadenectomy and radioiodine therapy were shown to be important independent prognostic factors. Despite the differences found in conditional survival probabilities between sex, age, tumor size, lymphadenectomy and radioiodine categories, estimates were also favorable for each stratum, with magnitudes ranging from 87.0% to 99.5%.

The greatest differences in survival were observed for patients with distant metastasis and for cases submitted to radiotherapy. As expected, individuals with IVC (TNM 7^th^ edition) or IVB (TNM 8^th^ edition) staging also had much lower survival. Thus, age ≥ 45 years (TNM 7^th^ edition), age ≥ 55 years (TNM 8^th^ edition), presence of distant metastasis and lymphadenectomy were considered as factors that negatively influenced the prognosis. On the other hand, radioiodine therapy was considered a protection factor.

Survival analysis has often been used to evaluate prognostic factors related to the patient and tumor, as well as to the efficacy of the treatments recommended for cancer. However, for DTC this evaluation is particularly difficult because of the low disease-related mortality rate. In this study, the probability of dying as a result of TC was 4.2% in 5 years. Mazzaferi et al.^14^ observed death probability at 4.0%, 6.0% and 8.0% at 10, 20, and 30 years, respectively, in a cohort of patients with DTC in the United States. Since their cohort was younger (mean age was 35.7 years), it could be a possible explanation for the observation of lower probability of death in the long-term.

As mentioned, the age ranges (< 45 years and ≥ 45 years) used by TNM 7^th^ edition^13^ was appropriate to predict death in the present study. However, the age ranges (< 55 years and ≥ 55 years) used by TNM 8^th^ edition^14^ seems to be better to stratify the risk of death. In fact, the patient’s age has been described in the literature^15^^,^^16^^–^^18^ as a relevant prognostic factor of death. The classic cut-off point was not observed in two hospital-based studies conducted in the United States^16^^–^^18^. Age above 50 years was described as an independent prognostic factor associated with death by DTC^16^. For PTC, another American study^18^ stratified age in three age groups (< 45 years, 45-60 years and > 60 years), using < 45 years as a reference category. This study showed an adjusted HR of 73.61 and 25.50 for the age groups of 45-60 and > 60 years, respectively. The possibility of death due to other causes before dying by TC was attributed for the reduced risk in those aged > 60 years.

Some studies argue that sex is a factor associated with death by TC^15^^,^^17^. Mazzaferi et al.^15^ evaluated the prognostic factors of DTC excluding cases with distant metastasis, and reported that women had a 50% lower risk of dying than men. McConahey el at.^17^ reported that, for PTC, male sex was highly associated with death. In contrast, sex did not influence the prognosis of PTC according Grogan et al.^18^. In our study, males presented a higher risk of death in the univariate analysis. However, the effect of this covariate could not be estimated in the multivariate analysis since it was observed that the baseline risk was not equal for women and men. To solve this non-proportionality, the survival modeling process was performed considering that sex divided the cohort into two strata with different baselines, and the effects of the other covariates being controlled by the variation in baseline risk attributable to men and women.

Our results – as well as in other studies^18^^,^^19^ – suggest that distant metastasis is an important prognostic factor for DTC. The estimates of death risk were very high, both in the univariate and in the multivariate analyses, whereas regional lymph node metastasis was not identified as a prognostic factor. In fact, few studies^18^^,^^19^ have considered that regional lymph node metastasis influences the survival of TC despite seeming to have a role in disease recurrence^15^^,^^18^^–^^23^. Although metastasis to regional lymph nodes did not impact the outcome, lymphadenectomy was surprisingly associated with increased risk of death. Perhaps the differences in the extent of lymphadenectomy may better explain these findings, and more information is needed to clarify them.

The benefits of radioiodine therapy for ablation of remaining thyroid tissue are controversial, especially in the early stages of the disease. For our study population, postoperative radioiodine therapy significantly improved DTC survival. Similarly, in the study of Mazzaferi et al.^15^, radioiodine therapy was a protective factor for differentiated non-metastatic carcinomas. For Jonklaas et al.^24^, a better specific survival was associated only in stages III and IV of radioiodine therapy. A similar finding was found by Carhill et al.^25^ in evaluating overall survival. However, Grogan et al.^18^ found no statistically significant association for PTC. Given the contradictory evidence in the literature, this therapy should not be used indiscriminately, especially in low risk patients^18^ given the highest risk of a second primary cancer^26^^,^^27^.

Tumor size (≥ 4.0 cm) and radiotherapy negatively influenced the outcome only in the univariate analysis. Contrary to what has been described in the literature^15^^,^^18^^,^^28^^–^^30^, in the present study, the DTC cohort survival did not differ according to the microcarcinoma, multifocal lesions and histological type (possibly due to the small proportion of FTC patients). Unfortunately, the risk of death related to the extension of surgery could not be evaluated in this study, since the proportion of cases submitted to subtotal thyroidectomy was very small.

Estimated survival from HBCR data tend to be higher than population-based cancer registries. Moreover, differences in eligibility criteria, outcome definition, classification of covariates, methods used to estimate survival and identify prognostic factors may interfere in the results obtained. The diagnostic criteria was changed in 1988^31^, and many cancers previously classified as follicular have come to be categorized as a follicular variant of papillary carcinoma, thus causing an increase in the proportion of PTC. Thus, studies that used data prior to this modification may have reached different conclusions.

Limitations of this study include retrospective data collection. However, conducting a prospective study to evaluate TC is very difficult due to the high survival rate of the disease. Because of few events, the low statistical accuracy makes the results hard to interpret. Furthermore, as this study covered a decade of data, some variation in the treatment may have occurred, making it difficult to compare the therapeutic protocols. Advances in diagnostic technologies have reduced the number of cases with tumor invasion to adjacent thyroid structures. Only three patients with non-resectable tumors were observed, and comparisons within this group were not possible. The lack of standard information in the histopathological reports made the evaluation of the subtypes of PTC and FTC impossible.

A very high survival over a 5-year period was found in this study. The prognosis was negatively influenced by age, distant metastasis and lymphadenectomy, whereas radioiodine therapy was found to be protective. When compared to TNM 7^th^ edition, the 8^th^ edition age ranges seems to be better to predict death in the present study.
